# Predictive value of dual-energy CT assessment of extracellular volume fraction on efficacy of immune checkpoint inhibitors in patients with liver cancer

**DOI:** 10.3389/fonc.2025.1659545

**Published:** 2025-10-22

**Authors:** Lixing Lei, Xiaohua Huang, Lingling Tang, Nian Liu, Ke Pan, Qianqian Liu

**Affiliations:** Affiliated Hospital of North Sichuan Medical College, Department of Radiology, Nanchong, China

**Keywords:** dual-energy CT, extracellular volume fraction, liver cancer, immune checkpoint inhibitors, cancer

## Abstract

**Objective:**

To investigate the predictive value of dual-energy CT assessment of extracellular volume fraction (ECV) on the efficacy of immune checkpoint inhibitors (ICIs) in patients with liver cancer.

**Methods:**

A retrospective analysis was conducted on 179 liver cancer patients who received ICIs in our hospital between November 2023 and February 2025. Patients were divided into a group with excellent treatment efficacy (n=103) and a group with poor treatment efficacy (n=76). Univariate and binary Logistics regression analyses were performed to identify factors influencing treatment efficacy. The predictive value of ECV for the efficacy of ICIs in liver cancer patients was evaluated with receiver operating characteristic (ROC) curve analysis.

**Results:**

There were statistically significant differences in ECV, CRP, IL-6, NEU, and the status of other anti-tumor treatments between the two groups (*P* < 0.05). Binary Logistics regression analysis indicated that ECV, CRP, IL-6, and NEU were factors influencing the efficacy of ICIs in liver cancer patients (*P* < 0.05). ROC analysis showed that ECV had the highest area under the curve (AUC) of 0.839, with a standard error of 0.029 (95% CI: 0.781-0.896), a Youden index of 0.54, sensitivity of 56.58%, specificity of 97.09%, and an optimal cutoff value of 0.45. Patients were grouped based on the optimal critical value of ECV, with the survival probability of patients with ECV ≤ 0.45 being higher than that of patients with ECV > 0.45, showing statistically significant differences (*P* < 0.05). Patients with ECV ≤ 0.45 also exhibited higher post-treatment immune function indicators compared to those with ECV > 0.45, with statistically significant differences (*P* < 0.05).

**Conclusion:**

Dual-energy CT assessment of ECV demonstrates good predictive value in the efficacy of ICIs in liver cancer patients, assisting clinical judgment of treatment efficacy.

## 1 Introduction

Liver cancer, as the third leading cause of cancer-related deaths globally (1), poses a serious threat to global public health due to its high incidence and mortality rates. The onset of liver cancer is closely associated with various risk factors, including hepatitis B virus (HBV) and hepatitis C virus (HCV) infections (2). While early-stage liver cancer can be effectively treated through surgical resection, local interventions, or liver transplantation, the majority of patients are diagnosed at advanced stages, and even with treatment, patients often experience recurrence or metastasis within five years. Therefore, systemic treatments play a crucial role in the management of liver cancer (3). In recent years, targeted therapies based on anti-angiogenic agents have become the primary choice for first-line treatment of advanced liver cancer. However, clinical studies (4) have shown that although drugs like sorafenib can prolong patient survival, their efficacy is limited and often accompanied by adverse events such as drug tolerance. This has prompted the medical community to continually explore new treatment modalities to enhance treatment outcomes and quality of life for liver cancer patients (5). With in-depth research into the tumor immune microenvironment and the interactions between immune cells and tumor cells, immune checkpoint inhibitors (ICIs) have emerged as a promising approach in cancer therapy. Specifically, drugs targeting programmed cell death protein 1 (PD-1) and its ligand programmed cell death ligand 1 (PD-L1) have demonstrated significant efficacy in the treatment of various solid tumors (6). In hepatocellular carcinoma, PD-L1 is primarily expressed on tumor cells, Kupffer cells, and hepatocytes, inhibiting T cell activity by binding to PD-1, thereby helping tumor cells evade immune system attacks. Therefore, the expression level of PD-L1 is closely associated with immune evasion, disease progression, and prognosis in liver cancer. Dual-Energy CT (DECT), as an advanced imaging technology, plays a crucial role in the diagnosis and assessment of treatment efficacy in tumors (7). It provides multidimensional information about tumor tissue structure and function, including key parameters such as extracellular volume fraction (ECV). ECV reflects the spatial proportion of the extracellular stroma in the tumor microenvironment, which is crucial for evaluating the degree of tumor fibrosis, metabolic status, and immune cell infiltration (8). Given the critical role of PD-L1 in immune evasion in liver cancer and the unique advantages of DECT in tumor assessment, this study aims to investigate the predictive value of DECT-assessed ECV on the efficacy of ICIs in patients with liver cancer. It is hoped that this research will provide new imaging-based evidence for personalized treatment of liver cancer patients, thereby optimizing treatment plans and improving treatment outcomes and quality of life for patients.

## 2 Materials and methods

### 2.1 Research objects

**Figure 1:**
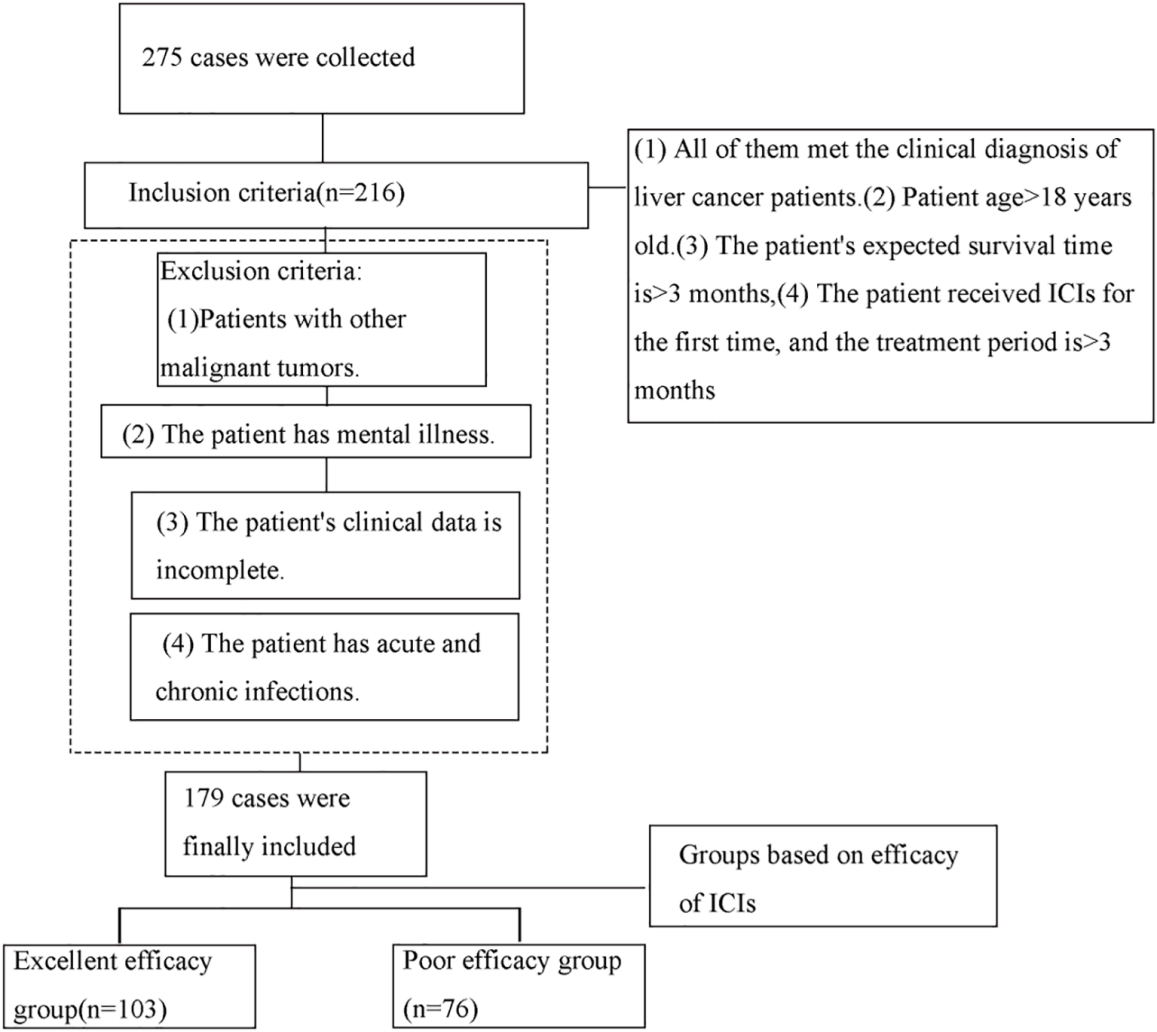
Flowchart.

A total of 179 liver cancer patients who received immune checkpoint inhibitors (ICIs) and were admitted to our hospital between November 2023 and February 2025 were retrospectively selected for this study. Patients were categorized into two groups based on the efficacy of ICIs: the good efficacy group (n=103) and the poor efficacy group (n=76). Criteria for classification were as follows: Patients were classified according to the RECIST criteria. Those achieving a complete response (disappearance of all target lesions with no new lesions) or a partial response (≥30% reduction in the sum of diameters of target lesions, with no new lesions) were assigned to the excellent efficacy group. Patients with stable disease (a change in the sum of target lesion diameters that did not meet the criteria for partial response [reduction <30%] or progressive disease [increase ≤20%]) and those with progressive disease (≥20% increase in the sum of diameters of target lesions or the appearance of new lesions) were assigned to the poor efficacy group. The inclusion criteria were as follows: (1) all patients met the clinical diagnosis of liver cancer; (2) age >18 years; (3) patients were expected to have a survival period of more than 3 months; (4) patients underwent their first ICIs treatment, and the treatment duration was over 3 months. The exclusion criteria were as follows: (1) patients with concurrent other malignancies; (2) patients with mental disorders; (3) patients with incomplete clinical data; (4) patients with acute or chronic infections. Refer to **Figure 1** for the screening process.

### 2.2 Research methods

Patient information and laboratory test data prior to treatment were collected through the electronic medical record system. This included: (1)General Information: gender, body mass index (BMI), liver cancer diameter, age, smoking history, underlying medical history (hypertension, diabetes), hepatitis B infection status, liver cancer type, immunotherapy regimen (monotherapy, combination therapy), and whether combined with other anti-tumor treatments. (2)CT Examination: Liver cancer examinations were conducted with the 3rd generation dual-source CT scanner from Siemens (SOMATOM Force) in Germany. Before the scan, patients were required to fast and abstain from water. Five to ten minutes before the scan, they were instructed to drink 800 to 1000 milliliters of water. The scan range extended from the diaphragm to the level of the navel. During the contrast-enhanced scan, iodixanol (320 mg/mL) was injected at high pressure via the antecubital vein at a rate of 2.2 to 3 mL/second, with a dose of 1.5 mL/kg body weight. The scan encompassed the arterial phase (25 to 30 seconds), portal venous phase (65 to 70 seconds), and equilibrium phase (2 minutes). Scan parameters were adjusted as needed, with real-time dynamic exposure modulation enabled. After the scan, images were uploaded to a workstation for further analysis. The Siemens post-processing workstation (syngo via) and Liver VNC program were used to analyze images from the equilibrium phase. Two experienced radiologists, blinded to the study, evaluated the results. If the intraclass correlation coefficient (ICC) for the measurements was greater than 0.8, the average of the two readings was taken. If the ICC was less than or equal to 0.8, a senior physician reevaluated the measurements. In lesion measurements, the average iodine concentration of three regions of interest (ROIs) on the lesion’s largest slice was calculated, avoiding necrotic and vascular areas. Iodine maps were generated with Liver VNC, and the iodine concentration of the lesion and adjacent main artery at the same level was measured to calculate the normalized iodine concentration (NIC). Combining the patient’s hematocrit (HCT) from the 48 hours before the examination, the extracellular volume fraction (ECV) was calculated with the formula ECV = NIC × (1 - HCT) (9). (3) Laboratory Parameters: Collection of pre-intervention white blood cell count (WBC), neutrophil count (NEU), lymphocyte count (LYM), blood urea nitrogen (BUN), creatinine (Cr); extracellular volume fraction (ECV), C-reactive protein (CRP), interleukin-6 (IL-6), and pre- and post-intervention immune indicators.

### 2.3 Statistical analysis

The experimental data collected were analyzed with SPSS 27.0 (International Business Machines Corporation, Armonk, New York, USA). The Shapiro-Wilk test was employed for normality testing. For normally distributed metric data, results were presented as `X ± S, and independent sample t-tests were used for comparisons. For non-normally distributed data, results were expressed using the median and interquartile range (IQR) as MQ2 (Q1, Q3). The Mann-Whitney U test was utilized for calculations in this case. Count data were presented as frequencies, and comparisons were conducted with χ^2^ test or Fisher’s exact test. Factors influencing the data were analyzed with univariate and binary Logistics regression analysis. The predictive value of ECV for the efficacy of ICIs in liver cancer patients was assessed with receiver operating characteristic (ROC) curve. A significance level of *P* < 0.05 was considered statistically significant.

## 3 Results

### 3.1 Univariate analysis of influencing factors

A comparison of ECV, CRP, IL-6, NEU, and the status of combined other anti-tumor treatments between the two groups of patients showed statistically significant differences (*P* < 0.05), as shown in **Table 1**.

**Table 1:** Univariate analysis of influencing factors.

Indicator	Classification	Poor Efficacy Group (n=76)	Good Efficacy Group (n=103)	*t/χ^2^/Z* Value	*P* Value
Age (years)		61.88 ± 8.43	61.96 ± 7.58	0.067	0.947
Gender	Male	42	60	0.159	0.690
	Female	34	43		
BMI (kg/m^2^)		21.15 ± 1.26	21.43 ± 3.17	0.728	0.467
Smoking	Yes	12	20	0.392	0.531
	No	64	83		
Comorbidities	Yes	21	36	1.080	0.299
	No	55	67		
HBV Infection	Yes	43	64	0.562	0.454
	No	33	39		
Liver Cancer Diameter (cm)	≤5	24	36	0.223	0.637
	>5	52	67		
Liver Cancer Type	Hepatocellular Carcinoma	56	84	1.589	0.207
	Intrahepatic Cholangiocarcinoma	20	19		
Immunotherapy Regimen	Monotherapy	74	102	0.575	0.387
	Combination Therapy	2	1		
Combined with other Anti-tumor Therapies	Yes	52	64	5.543	0.019
	No	14	40		
ECV		0.46(0.36,0.55)	0.32(0.27,0.40)	-7.739	<0.001
CRP (mg/L)		5.62 ± 1.18	4.49 ± 0.91	7.234	<0.001
IL-6 (pg/mL)		6.18 ± 1.29	5.33 ± 1.07	4.811	<0.001
WBC (×10^10^/L)		6.71 ± 3.28	6.46 ± 2.22	0.608	0.544
LYM (×10^9^/L)		0.96 ± 0.19	0.98 ± 0.20	0.675	0.500
NEU (×10^9^/L)		5.22 ± 0.80	4.49 ± 0.68	6.584	<0.001
BUN (mmol/L)		4.11 ± 1.09	4.22 ± 1.37	0.578	0.564
Cr (μmol/L)		81.08 ± 15.29	79.99 ± 16.41	0.452	0.652
PLT (×10^9^/L)		127.62 ± 25.95	133.21 ± 26.41	1.410	0.160

BMI, Body Mass Index; WBC, White Blood Cell Count; NEU, Neutrophil Count; LYM, Lymphocyte Count; BUN, Blood Urea Nitrogen; Cr, Creatinine; EVC, Extracellular Volume Fraction; CRP, C-Reactive Protein; IL-6 Interleukin-6.

### 3.2 Binary logistics regression analysis of influencing factors

Using the variables identified as significant in the univariate analysis as independent variables, a binary Logistics regression analysis was conducted with the efficacy of ICIs as the dependent variable (poor = 1, good = 0). The results of the binary logistic regression analysis indicated that ECV, CRP, IL-6, and NEU were influencing factors for the efficacy of ICIs in patients with liver cancer (*P* < 0.05), as shown in **Tables 2**, **3**.

**Table 2:** Variable assignment.

Influencing factors	Assignment
ECV	Original Value
CRP	Original Value
IL-6	Original Value
NEU	Original Value
Combined with other Anti-tumor Therapies	No=0, Yes=1

**Table 3:** Binary logistics regression analysis results.

Variable	β	Standard error	Wald	P	Exp(β)	95%CI		Collinearity diagnostics	
						Lower limit	Upper limit	Tolerance	VIF
ECV	1.068	0.257	17.276	<0.001	2.910	1.759	4.816	0.849	1.178
CRP	0.921	0.238	14.959	<0.001	2.511	1.575	4.004	0.849	1.177
IL-6	0.906	0.357	6.429	0.011	2.474	1.228	4.982	0.961	1.040
NEU	0.525	0.543	0.937	0.333	1.691	0.584	4.901	0.807	1.240
Combined with other Anti-tumor Therapies	1.777	0.332	28.576	<0.001	5.914	3.082	11.347	0.974	1.027
Constant	-22.624	3.625	38.954	<0.001	<0.001	–	–	–	–

### 3.3 ROC curve analysis of predictive value of indicators

**Figure 2:**
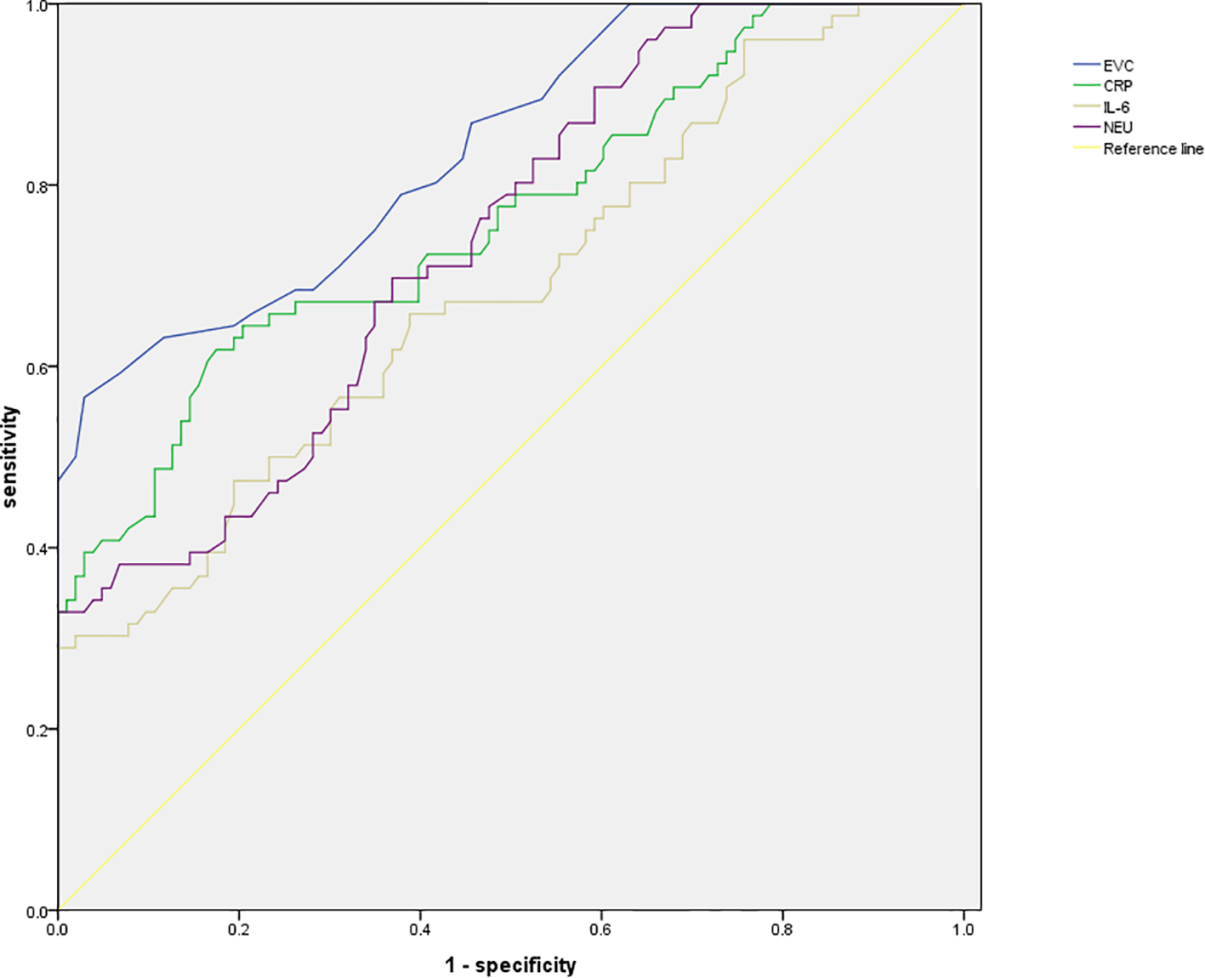
ROC curve.

The ROC analysis results revealed that the area under the curve (AUC) for ECV was the highest at 0.839, with a standard error of 0.029 (95% CI: 0.781-0.896) and a Youden index of 0.54. At this point, the sensitivity was 56.58% and the specificity was 97.09%, with the optimal cutoff value being 0.45. See **Table 4**, **Figure 2** for details.

**Table 4:** ROC analysis results.

Indicators	AUC	SE	95%CI	Youden	Sensitivity	Specificity	*P* value	Optimal cutoff value
ECV	0.839	0.029	0.781~0.896	0.54	56.58	97.09	<0.001	0.45
CRP	0.764	0.036	0.693~0.835	0.44	61.84	82.52	<0.001	5.49
IL-6	0.684	0.040	0.605~0.763	0.30	68.95	61.54	<0.001	7.15
NEU	0.739	0.036	0.667~0.810	0.35	72.85	62.16	<0.001	5.68

### 3.4 Optimal cutoff value of ECV and patient survival rate

This study was followed up for 1 year, patients were grouped based on the optimal cutoff value of ECV. The group with ECV ≤ 0.45 exhibited a higher probability of survival compared to the group with ECV > 0.45, with statistically significant differences (*P* < 0.05), as shown in **Table 5**.

**Table 5:** Optimal cutoff value of ECV and patient survival rate.

Item	ECV ≤ 0.45 (n=139)	ECV>0.45 (n=40)	*χ (*2 )value	*P* value
Survival Rate	87.05	70.00	6.473	0.011
Survival	121	28		
Death	18	12		

### 3.5 Comparison of immunological indicators before and after treatment in patients with different ECV values

Patients in the group with ECV ≤ 0.45 showed higher levels of immunological indicators after treatment compared to the group with ECV > 0.45, with statistically significant differences (*P* < 0.05), as illustrated in **Table 6**.

**Table 6:** Comparison of immunological indicators before and after treatment in patients with different ECV values (%).

Indicators	Classification	ECV ≤ 0.45 (n=139)	ECV>0.45 (n=40)	*χ (*2) value	*P* value
CD4^+^T-LYM	Before Treatment	32.49 ± 3.19	32.59 ± 3.24	0.174	0.862
	After Treatment	37.68 ± 2.66	35.35 ± 2.41	4.981	<0.001
CD8^+^T-LYM	Before Treatment	27.08 ± 2.94	27.11 ± 2.94	0.057	0.955
	After Treatment	29.78 ± 2.87	28.51 ± 2.06	2.610	0.010
CD4^+^/CD8^+^T-LYM	Before Treatment	1.20 ± 0.29	1.19 ± 0.27	0.195	0.846
	After Treatment	1.34 ± 0.16	1.22 ± 0.17	4.122	<0.001

## 4 Discussion

This study focused on the predictive value of ECV assessed by dual-energy CT for the efficacy of ICIs in patients with liver cancer. Through the retrospective selection of data from 179 liver cancer patients who received ICIs, our analysis and exploration revealed that ECV holds a certain value in predicting the efficacy of ICIs in liver cancer patients.

The results of this study indicate that patients in the poor efficacy group had significantly higher ECV values compared to the good efficacy group, suggesting that high ECV may be associated with an unfavorable immune microenvironment in patients with liver cancer. Previous studies by Ozaki et al. (10) also demonstrated a certain discriminatory value of ECV in patients with viral hepatitis, related normal liver, and chronic liver diseases. Patients with liver diseases tended to have higher ECV values, aligning closely with the findings of this study. The analysis suggests that ECV reflects the spatial proportion of the extracellular matrix in the tumor microenvironment, which is closely linked to the immune microenvironment of liver cancer (11). During the development of liver cancer, the tumor microenvironment undergoes complex changes, including extracellular matrix remodeling, immune cell infiltration, and cytokine secretion (12, 13). An increase in ECV may indicate excessive deposition of the extracellular matrix, which could hinder the infiltration of immune cells into the tumor tissue, affecting the interaction between immune cells and tumor cells. High ECV may create a suppressive immune microenvironment, making it challenging for immune checkpoint inhibitors to exert their full effects (14). For instance, an excess of the extracellular matrix could envelop tumor cells, forming a physical barrier that prevents immune cells from identifying and attacking tumor cells. Additionally, certain components within the extracellular matrix may secrete inhibitory cytokines, further suppressing the activity of immune cells (15). Therefore, ECV serves as an important indicator reflecting the tumor microenvironment and may have a potential association with the efficacy of ICIs in patients with liver cancer.

Through binary Logistics regression analysis, this study identified ECV as one of the influencing factors for the efficacy of ICIs in patients with liver cancer. The underlying mechanisms behind this finding may be related to the regulation of immune cell activity and function. The mechanism of action of immune checkpoint inhibitors primarily involves such blocking immune checkpoint pathways as PD-1/PD-L1, activating T cells, and enhancing their cytotoxic effects on tumor cells (16). However, when ECV is abnormal in the tumor microenvironment, it may affect the activity and function of immune cells. High ECV could disrupt the metabolism and function of immune cells. Changes in the extracellular matrix may impact the ability of immune cells to acquire nutrients and oxygen, thereby affecting their proliferation and differentiation. Moreover, high ECV may also influence the expression and function of surface receptors on immune cells, leading to reduced responsiveness of immune cells to immune checkpoint inhibitors (17). For example, the expression and function of PD-1 on T cells may be affected by components of the extracellular matrix, making it challenging for immune checkpoint inhibitors to effectively block the PD-1/PD-L1 pathway, thereby impacting the efficacy of ICIs. On the other hand, changes in ECV may be associated with the biological behavior of tumor cells. High ECV may indicate that tumor cells have increased invasiveness and metastatic potential. These tumor cells may further modulate the immune microenvironment and evade immune surveillance by secreting various cytokines and growth factors (18). Therefore, as a critical feature of the tumor microenvironment, ECV can influence the activity and function of immune cells through various pathways, subsequently affecting the efficacy of ICIs in patients with liver cancer. In HCC research, ECV is closely linked to the regulation of the immune microenvironment. The immune microenvironment of HCC is complex and contains a variety of immune cells and cytokines, which interact to affect tumor progression. As mentioned in the previous research (19), many genes, such as KIF2C, CDK1, etc., not only participate in processes such as cell cycle regulation, but may also regulate the immune microenvironment. For example, abnormal expression of certain genes may alter antigen presentation on the surface of tumor cells and affect the recognition and killing functions of immune cells. These genes also hold significance in predicting responses to ICIs. ICI activates the immune system to fight tumors by blocking immune checkpoints. Studies found that (20) specific gene expression patterns were associated with ICI treatment effectiveness. The expression level of the genes screened through bioinformatics analysis may serve as a biomarker to predict ICI response. High expression of certain genes may indicate that patients respond well to ICI treatment; otherwise, they may respond poorly (21). In-depth exploration of the relationship between ECV-related factors, the immune microenvironment, and ICI response will enable more accurate prediction of ICI efficacy, support the development of personalized treatment strategies for HCC patients, enhance therapeutic outcomes, and ultimately improve patient prognosis.

Further predictive value analysis was conducted, and the ROC results revealed that the AUC for ECV was 0.839, with a standard error of 0.029, a 95% CI of 0.781 - 0.896, and a Youden index of 0.54. At this threshold, the sensitivity was 56.58%, and the specificity was 97.09%, with the optimal cutoff value being 0.45. These findings indicate that ECV exhibits a high level of accuracy in predicting the efficacy of ICIs in patients with liver cancer. Accurately predicting the efficacy of ICIs in liver cancer patients is crucial in clinical practices. Through the assessment of ECV using dual-energy CT, physicians can stratify patients before treatment, identifying those who may respond better to ICIs and thus tailor more personalized treatment plans. For patients with ECV ≤ 0.45, due to their higher probability of survival and improved immunological indicators after treatment, a more proactive approach with ICIs treatment could be considered, possibly in combination with other anti-tumor therapies to enhance treatment outcomes. For patients with ECV > 0.45, physicians may contemplate adjusting treatment plans, such as combining different treatment modalities or utilizing immunotherapeutic drugs with distinct mechanisms of action to improve patient outcomes. Additionally, a study by Bak et al. (22) demonstrated an independent association between ECV derived from dual-energy CT and the presence of liver dysfunction, aligning closely with the findings of this study, further confirming the relevance of ECV in liver cancer. This underscores the importance of ECV assessment as a valuable reference indicator for clinical researches. In clinical trials, grouping patients based on ECV values can enhance the accuracy and reliability of study outcomes. Furthermore, conducting in-depth investigations into the relationship between ECV and the efficacy of ICIs can help identify novel treatment targets and intervention strategies, offering new insights and approaches for the treatment of liver cancer. Finally, previous studies have shown (23) that ECV, as an imaging biomarker for the prognosis and treatment response of liver cancer, has important clinical utility in patients with liver cancer. The high incidence of hepatocellular cancer in men is related to factors such as testosterone, dihydrotestosterone, androgen receptor and proteomic defects. Current treatment strategies, including chemotherapy, remain insufficient, highlighting the urgent need for novel therapeutic approaches. Cyclic peptides have shown potential in the design of anti-liver cancer drugs. In this context, ECV can provide key information for prognosis assessment of liver cancer patients, assist in judging treatment response, and help doctors adjust treatment plans in a timely manner. It is of great significance in improving patient prognosis and exploring more effective treatment strategies (24). In this ROC analysis, the AUC of ECV reached 0.839, indicating that it has certain predictive power and a specificity of 97.09%, which means that most liver cancer patients who have poor response to immune checkpoint inhibitors can be accurately excluded and unnecessary treatment can be avoided. However, the sensitivity is only 56.58%, which means that nearly half of the actual effective patients will be missed, affecting clinical application. In the future, the sample size can be expanded to include patients with different characteristics, so that the model can learn more comprehensive information. In addition, integrating ECV with other biomarkers for multi-dimensional evaluation may enhance the identification of responsive patients, thereby improving sensitivity while maintaining high specificity and reducing the trade-off between the two.

While this study has made significant progress, there are several limitations that should be acknowledged. Firstly, being a retrospective study, there may be such inherent biases as selection bias and information bias. The process of patient selection could have been influenced by various factors, resulting in the study sample not fully representing all patients with liver cancer. Secondly, this study was conducted at a single center with a relatively limited sample size, which could affect the generalizability and reliability of the study results. In addition, this study has limitations related to potential confounding effects from combined antitumor therapies. For instance, the inclusion of “combination with other anti-tumor therapies” may influence the efficacy evaluation. Although hierarchical analysis and multivariate regression could help account for these factors, the complex mechanisms of action of different combination regimens may still interfere with the accurate correlation between extracellular volume fraction and immune efficacy. Future research could aim to expand the sample size and conduct multi-center, prospective studies to enhance the accuracy of research findings. With the continuous development of imaging technology and immunotherapy, the application prospects of dual-energy CT for evaluating ECV in liver cancer treatment are promising. Future researches could delve deeper into exploring the relationship between ECV and the tumor microenvironment, immune cell function, and the efficacy of immunotherapy. This could help uncover the value of ECV in early diagnosis, treatment monitoring, and prognosis assessment of liver cancer. Additionally, by integrating other imaging techniques and biological markers, a more comprehensive and accurate predictive system could be established to provide a more scientific basis for personalized treatment of liver cancer patients. Furthermore, based on the research outcomes related to ECV, targeted interventions could be developed, such as modulating the composition and function of the extracellular matrix to improve the tumor microenvironment and enhance the efficacy of immune checkpoint inhibitors. These interventions could potentially bring new hope for the treatment of liver cancer patients.

## 5 Conclusion

In conclusion, dual-energy CT assessment of ECV demonstrates good predictive value in the efficacy of ICIs in patients with liver cancer. ECV is closely associated with the immune microenvironment of liver cancer, impacting the activity and function of immune cells through various pathways, thereby influencing the efficacy of ICIs. In clinical practices, evaluating ECV can assist physicians in assessing patient response and developing more personalized treatment plans. Despite the limitations of this study, it has laid an important foundation and provided direction for subsequent research. Future studies should focus on further exploring the mechanisms and clinical applications of ECV, with the aim of providing more effective approaches for liver cancer treatment.

## Data Availability

The raw data supporting the conclusions of this article will be made available by the authors, without undue reservation.
